# Signal One and Two Blockade Are Both Critical for Non-Myeloablative Murine HSCT across a Major Histocompatibility Complex Barrier

**DOI:** 10.1371/journal.pone.0077632

**Published:** 2013-10-17

**Authors:** Kia J. Langford-Smith, Zara Sandiford, Alex Langford-Smith, Fiona L. Wilkinson, Simon A. Jones, J. Ed Wraith, Robert F. Wynn, Brian W. Bigger

**Affiliations:** 1 Stem Cell & Neurotherapies, Institute of Human Development, Faculty of Medical and Human Sciences, University of Manchester, Manchester, United Kingdom; 2 Genetic Medicine, St Mary’s Hospital, Manchester, United Kingdom; 3 Blood and Marrow Transplant Unit, Royal Manchester Children’s Hospital, Manchester, United Kingdom; Beth Israel Deaconess Medical Center, Harvard Medical School, United States of America

## Abstract

Non-myeloablative allogeneic haematopoietic stem cell transplantation (HSCT) is rarely achievable clinically, except where donor cells have selective advantages. Murine non-myeloablative conditioning regimens have limited clinical success, partly through use of clinically unachievable cell doses or strain combinations permitting allograft acceptance using immunosuppression alone. We found that reducing busulfan conditioning in murine syngeneic HSCT, increases bone marrow (BM):blood SDF-1 ratio and total donor cells homing to BM, but reduces the proportion of donor cells engrafting. Despite this, syngeneic engraftment is achievable with non-myeloablative busulfan (25 mg/kg) and higher cell doses induce increased chimerism. Therefore we investigated regimens promoting initial donor cell engraftment in the major histocompatibility complex barrier mismatched CBA to C57BL/6 allo-transplant model. This requires full myeloablation and immunosuppression with non-depleting anti-CD4/CD8 blocking antibodies to achieve engraftment of low cell doses, and rejects with reduced intensity conditioning (≤75 mg/kg busulfan). We compared increased antibody treatment, G-CSF, niche disruption and high cell dose, using reduced intensity busulfan and CD4/8 blockade in this model. Most treatments increased initial donor engraftment, but only addition of co-stimulatory blockade permitted long-term engraftment with reduced intensity or non-myeloablative conditioning, suggesting that signal 1 and 2 T-cell blockade is more important than early BM niche engraftment for transplant success.

## Introduction

Haematopoietic stem cell transplantation (HSCT) is used to treat several genetic disorders, where a diffusible factor delivered by donor cells can complement the disease. Both the dose of protein or enzyme delivered by donor cells and the level of donor chimerism achieved are important to achieve maximal correction, as illustrated in the lysosomal disease Mucopolysaccharidosis I (MPS I) Hurler [1]. HSCT is usually limited to life-threatening genetic disorders due to the risks associated with myeloablative conditioning (MAC) regimens required to prevent transplant rejection. To expand the application of HSCT to broader indications, such as attenuated diseases, reduced intensity conditioning (RIC) or non-myeloablative conditioning (NMC) would be preferred, but this can lead to transplant rejection or low donor chimerism [1,2]. 

Graft rejection involves multiple mechanisms [3], but the most widely used target in RIC is the T cell. Numerous RIC regimens for allogeneic HSCT targeting the T cell have been determined in mice (Table 1), but their clinical applicability has been limited, partly due to determination of mouse regimens in non-stringent transplant settings [4-6], and others have been determined using clinically unachievable cell doses [7-10]. Non-depleting anti-CD4 and anti-CD8 monoclonal antibodies (mAbs) with anti-CD40L costimulation blockade achieved 25-40% donor chimerism using moderate cell doses (20x10^6^), but only in permissive strain combinations, whilst C57BL/6 recipients are resistant to this method of transplant tolerance generation [5,6,11]. In more stringent allo-transplant models using C57BL/6 recipients and MHC mismatched donor cells, rejection is often overcome using high cell doses and/or some myeloablation. T cell depleting anti-CD4 and anti-CD8 mAbs with very high cell doses (200x10^6^) and 7Gy thymic irradiation (TI) can achieve 20-35% donor chimerism, but only 10-15% if 3.5Gy is used [7]. In other models these mAbs are combined with myeloablative chemotherapy agents, such as busulfan, which is an alkylating agent with specific action against primitive haematopoietic stem cells (HSCs) [12,13], and immune supressing agents such as sirolimus (rapamycin), which prevents the action of T and B cells by blocking cytokine receptors for IL-2 [14]. Combining these mAbs with 20-40mg/kg busulfan, moderate cell doses (25-40x10^6^) and sirolimus can generate 60-80% donor chimerism, but only 10-30% with lower non-myeloablative busulfan doses [15,16]. Costimulation blockade with anti-CD40L mAb and occasionally CTLA4Ig is often combined with 3Gy total body irradiation (TBI), generating 5-80% donor chimerism in C57BL/6 recipients with moderate cell doses (20-40x10^6^) [15,17-20]. Further reduction of TBI in combination with anti-CD40L reduces chimerism [21], whilst addition of donor specific transfusion (DST) does not lead to significant increases [22,23]. Regimens with immune suppression but no myeloablation all use high cell doses (50-200x10^6^), and resulting donor chimerism (1-40%) is lower than regimens involving myeloablation [4,8,9,24]. These studies show that myeloablation is important in achieving high donor chimerism, but the relatively high levels of myeloablation and the permissive conditions used to achieve them make these regimens an unattractive clinical proposition. In the clinic, patients often receive a cell dose that is only just sufficient for repopulation [25], therefore it is unfeasible to overcome graft rejection using high cell doses. 

**Table 1:** Examples of reduced intensity conditioning regimens in mouse models of allogeneic HSCT using antibodies against CD4, CD8 or CD40L.

	**Donor**	**Recipient**	**Myeloablation**	**mAb**	**Additional treatment**	**Cell dose**	**Chimerism^a^**
**Depleting anti-CD4/CD8 mAbs**	B10.A	C57BL/6	3.5 or 7Gy TI	Anti-CD4 (1.8mg) and anti-CD8 (1.4mg), days -5, 1, 7	-	200x10^6^	10-15% or 20-35% [7]
	Balb/c	C57BL/6	20mg/kg busulfan	Anti-CD4 and anti-CD8 (0.25mg), days -3 to -1	Sirolimus (3mg/kg), days 0-14	40x10^6^	~80% [15]
	Balb/c	C57BL/6	5, 10, 20 or 40mg/kg busulfan, day -3	Anti-CD4 and anti-CD8 (0.2mg), days -9, -5, -2, 0, 2, 7	Sirolimus (24mg/kg), day -1	25x10^6^	10, 30, 60 or 80% [16]
**Costimulation blockade**	B10.A	C57BL/6	3Gy TBI	Anti-CD40L (2mg), day 0	-	20x10^6^	~65%**^b^** [17]
	B10.A	C57BL/6	3Gy TBI	Anti-CD40L (2mg), day 0	T cell depleted donor BMCs	20x10^6^	20-80%^b^ [18]
	Balb/c	C57BL/6	3Gy TBI	Anti-CD40L (0.5mg), days 0, 2, 4, 6	-	40x10^6^	~60% [15]
	Balb/c	C57BL/6	200cGy TBI	Anti-CD40L (200ug), days -1 to 5, twice weekly to day 14	-	40x10^6^	~48% [21]
	Balb/c	C57BL/6	3Gy TBI	Anti-CD40L (1mg), day 0, CTLA4Ig (0.5mg), day 2	-	20x10^6^	30-70% [19], 5-45% [20]
	Balb/c	B6.SJL-Ptprc^a^Pep3^b^	3Gy TBI	Anti-NK1.1 (0.5mg), day -3, Anti-CD8a (0.5mg), day -2, Anti-CD40L (0.5mg), day 0	175mg/kg cyclophosphamide	30x10^6^	~60% [46]
	B10.A	C57BL/6	3Gy TBI	Anti-CD40L 2mg day 0	10x10^6^ donor splenocytes, day -7	20x10^6^	~60%^b^ [22]
	B6.SJL	Balb/c	100cGy TBI	Anti-CD40L (1.6mg), days -10, -7, -3, 0, 3	10x10^6^ donor splenocytes, day -10	40x10^6^	~25% [23]
	Balb/c	C57BL/6	30mg/kg busulfan, day -1	Anti-CD40L (0.5mg), days 0, 4, Anti-LFA-1 (0.1mg), days 0, 2, 4	Or Everolimus (3mg/kg), day 0-8, Or DSG (4mg/kg), day 0-8	20x10^6^	30-100% [26]
	Balb/c	C57BL/6	-	Anti-CD40L (0.5mg), day 0, Anti-NK1.1 (0.25mg), days -5, -1	-	30x10^6,^ 100x10^6^	0-5%, 1-40% [8]
	C57BL/6	Balb/c, CBA, B10.BR	-	Anti-CD40L (0.5mg), days -7, -4, 0, 3	10x10^6^ donor splenocytes, day -7	50x10^6^	~8, 9, 17% [4], ~10% [24]
	Balb/c	C57BL/6	-	Anti-CD40L (1mg), day 0, CTLA4Ig (0.5mg), day 2	Rapamycin (0.2mg/kg/day), Methylprednisolone (10mg/kg/day), Mycophenolate mofetil (20mg/kg/day), For 4 weeks after HSCT	50x10^6^, 100x10^6^ 200x10^6^	0-5%, 1-15%, 5-30% [9]
	B10.A	C57BL/6	-	Anti-CD40L (0.5mg), day 0, CTLA4Ig (0.5mg), day 2	-	200x10^6^	2-12% [10]
**Non-depleting anti-CD4/CD8 + costimulation blockade**	BL10.BR or C57BL/10	CBA	-	Anti-CD4 and anti-CD8 (1mg), days -28, -26, -24, 0, 2, 4, Anti-CD40L (1mg), days 0, 2, 4	T cell depleted donor BMCs	20x10^6^	~25% [6], ~40% [5]
	C57BL/10	CBA	-	Anti-CD4, anti-CD8 and anti-CD40L (1mg), days -28, -26, -24, 0, 2, 4	Skin graft, day -28 or 0, T cell depleted donor BMCs	40x10^6^	~30%^c^ [11]

^a^ Chimerism is the furthest reported, ranging between 6 and 50 weeks post-transplant, only 4 studies reported to <12 weeks, half reported to > 20 weeks post-transplant. Single values represent the mean donor chimerism in peripheral blood, unless otherwise indicated. Where total donor chimerism was not available, chimerism is given as a range representing the values reported for different lineages. ^b^ B cell chimerism only. ^c^ T cell chimerism only.

In summary, few RIC protocols for murine allogeneic HSCT achieve >80% donor chimerism, and those that do include moderate myeloablation, multiple immune suppressing agents and high donor cell doses [15,16,18,26]. In addition, strain combinations where immune suppression alone can result in allo-transplant acceptance are used, which is in contrast to the clinical scenario where both myeloablation and immunosuppression are required to achieve allo-transplant engraftment [3]. Lastly, significant long-term donor chimerism using NMC across an MHC barrier with clinically relevant cell doses has not been achieved to date, whilst the factors required to achieve this remain elusive. 

In order to improve the stringency of existing allo-transplant regimens we therefore developed a fully MHC mismatched mouse model of HSCT using low CBA donor cell doses into C57BL/6 recipients. This model requires fully myeloablative busulfan conditioning combined with T cell co-receptor blockade of signal 1 for long-term graft acceptance. Having identified that higher syngeneic chimerism in C57BL/6 recipients was associated with an increased ratio of donor to recipient haematopoietic cells in BM initially after transplant, we then compared published methods for improving donor to recipient cell number in the BM niche including ACK2 [27], G-CSF [28] or high cell dose [7], against further costimulatory blockade of signal 2 [5], all in combination with RIC and signal 1 blockade in our CBA-C57BL/6 transplant model. Despite early engraftment with G-CSF or high cell doses, costimulatory blockade was the only factor that could permit the use of NMC in combination with signal 1 blockade in this stringent mouse model of transplantation.

## Results

### Syngeneic engraftment is influenced by the early ratio of donor: recipient cells in the BM niche after transplant

**Figure 1:**
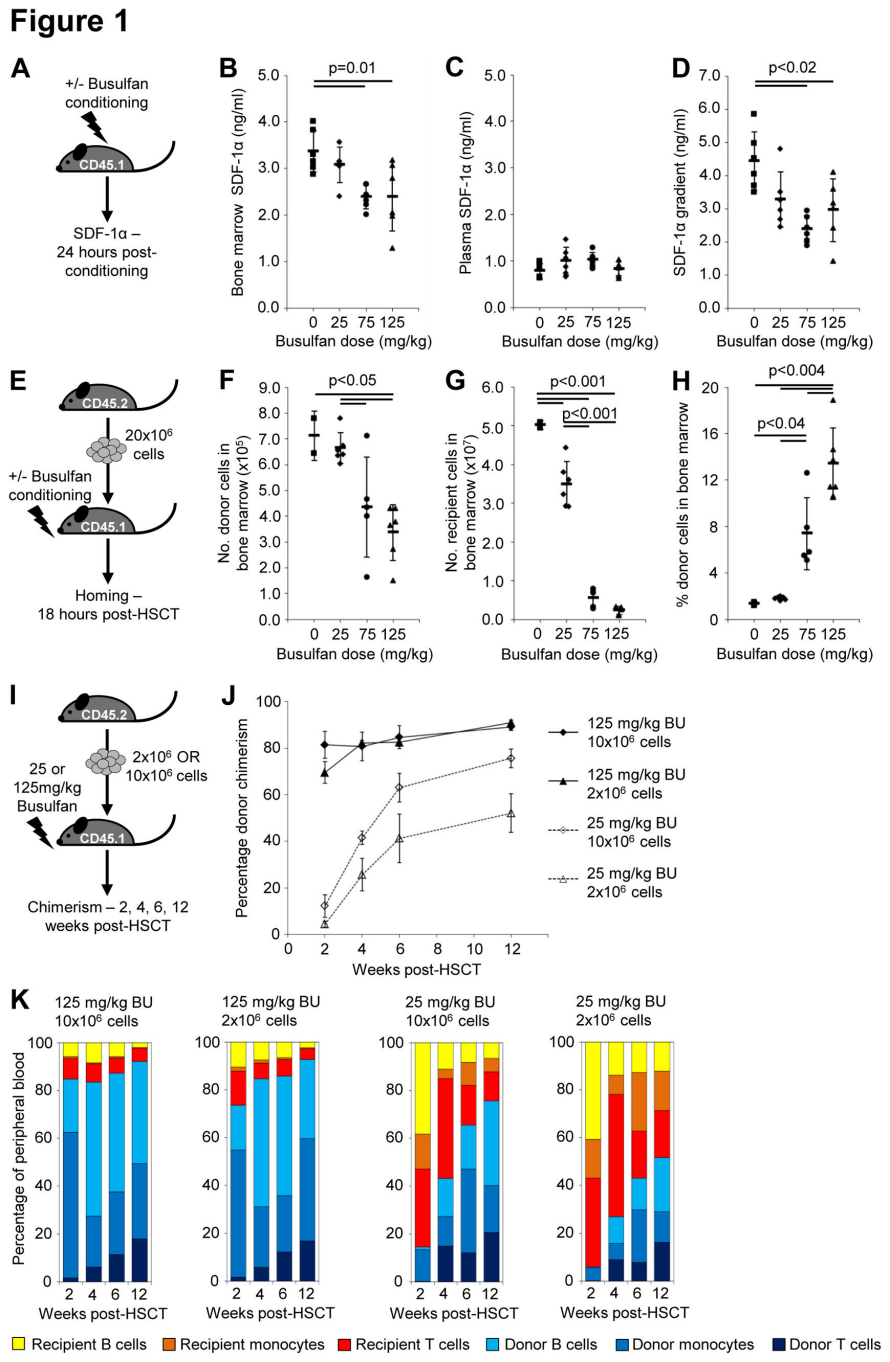
Syngeneic engraftment is influenced by the early ratio of donor: recipient cells in bone marrow after transplant. (**A**) Mice were conditioned with 0, 25, 75 or 125mg/kg busulfan; the concentration of SDF-1α was measured in (**B**) bone marrow and (**C**) plasma, 24 hours after the last dose of busulfan was administered (n=6 per group). (**D**) The SDF-1α gradient between bone marrow and plasma was calculated by dividing the bone marrow SDF-1α concentration by the plasma SDF-1α concentration. (**E**) Syngeneic HSCT with 20x10^6^ cells (CD45.2) was performed in recipients (CD45.1) that had received 0, 25, 75 or 125mg/kg busulfan (n=2-6 per group). (**F**) The number of donor and (**G**) recipient cells and (**H**) the percentage of donor cells in the bone marrow at 18 hours post-transplant were detected using flow cytometry. In B, C, D, F G and H each data point represents one recipient, horizontal bars are the mean and error bars represent the standard deviation. (**I**) HSCT recipients (CD45.1) were treated with 125mg/kg or 25mg/kg busulfan (BU) before receiving 2x10^6^ or 10x10^6^ syngeneic (CD45.2) donor bone marrow cells (n=5 per group). Donor chimerism in peripheral blood was monitored using flow cytometry at 2, 4, 6, and 12 weeks post-transplant. (**J**) The mean percentage donor chimerism over time is shown; error bars represent the standard deviation. (**K**) The mean percentage contribution of donor and recipient T cells, monocytes and B cells over time is also shown, as a percentage of the peripheral blood.

We chose to develop RIC regimens using busulfan, because it is considered less toxic than irradiation; it is myeloablative but not immune suppressive and has specific action against primitive HSCs [12,13]. It is used in many clinical transplant regimens for genetic diseases [2]. We initially sought to determine the impact of busulfan conditioning on migration of donor cells to the BM niche. SDF-1/CXCR4 is the major axis of donor cell migration to the BM [29]; therefore we determined the effect of busulfan dose on BM and plasma concentration of SDF-1α (Figure 1A). Mice receiving 75 and 125mg/kg busulfan had lower BM SDF-1α than mice receiving 0 or 25mg/kg (Figure 1B), whilst plasma SDF-1α was not affected (Figure 1C). As a result, the BM: plasma SDF-1α gradient is reduced with 75 and 125mg/kg busulfan, but is statistically indistinguishable with 0 and 25mg/kg busulfan (Figure 1D). 

To determine the effect of these altered SDF-1α levels on donor cell migration to the BM, we performed syngeneic HSCT and determined the number and percentage of donor cells that homed to BM (Figure 1E). Significantly fewer donor cells reached the BM in mice receiving 125mg/kg busulfan compared to either 0 or 25mg/kg, and in mice receiving 75mg/kg busulfan compared to 25mg/kg, with about half as many donor cells reaching the BM in the higher dose groups (Figure 1F). However, the number of recipient cells present in the BM is also significantly reduced in mice receiving busulfan, particularly at doses of 75 and 125mg/kg (Figure 1G), therefore the ratio of donor: recipient cells in BM is increased in mice receiving higher busulfan doses (Figure 1H), due to greater ablation of recipient cells. Recipients of 0 or 25mg/kg busulfan have only 2% donor cells in BM, whilst mice receiving 75mg/kg or 125mg/kg busulfan have significantly greater donor chimerism of 4-12% and 10-20% respectively (Figure 1H). 

We then compared long-term engraftment in mice receiving either NMC with 25mg/kg busulfan or MAC with 125mg/kg busulfan [30] (Figure 1I), which we have confirmed is equivalent in C57BL/6 recipients to 10Gy TBI and generates full donor chimerism after transplant of 2x10^6^ syngeneic BM cells [31] (Figures S1A,B). Myeloablative busulfan conditioning results in 70-80% donor chimerism after 2 weeks, rising to >90% by 12 weeks post-transplant (Figure 1J). Conditioning with 25mg/kg busulfan also results in long-term donor chimerism, although the level of engraftment is reduced. Recipients of 2x10^6^ cells have average donor chimerism of 4% after 2 weeks, which increases to 52% by 12 weeks post-transplant, whilst recipients of 10x10^6^ cells have 12% after 2 weeks, and 76% by 12 weeks post-transplant (Figure 1J). In mice receiving 25 or 125mg/kg busulfan, the percentage contribution of different lineages to peripheral blood demonstrates that at 2 weeks post-transplant the donor population consists mainly of monocytes, and as the donor population expands the contribution of T cells and B cells increases, whilst recipient T cells and B cells are reduced (Figure 1K).

Therefore reduced SDF-1α associated with high dose busulfan does reduce homing, but in syngeneic transplant the amount of recipient cell ablation, and thus the percentage of donor cells in the BM niche is more important in determining long-term engraftment levels. 

### Treatments that increase initial allogeneic chimerism are not sufficient for long-term engraftment with RIC

**Figure 2:**
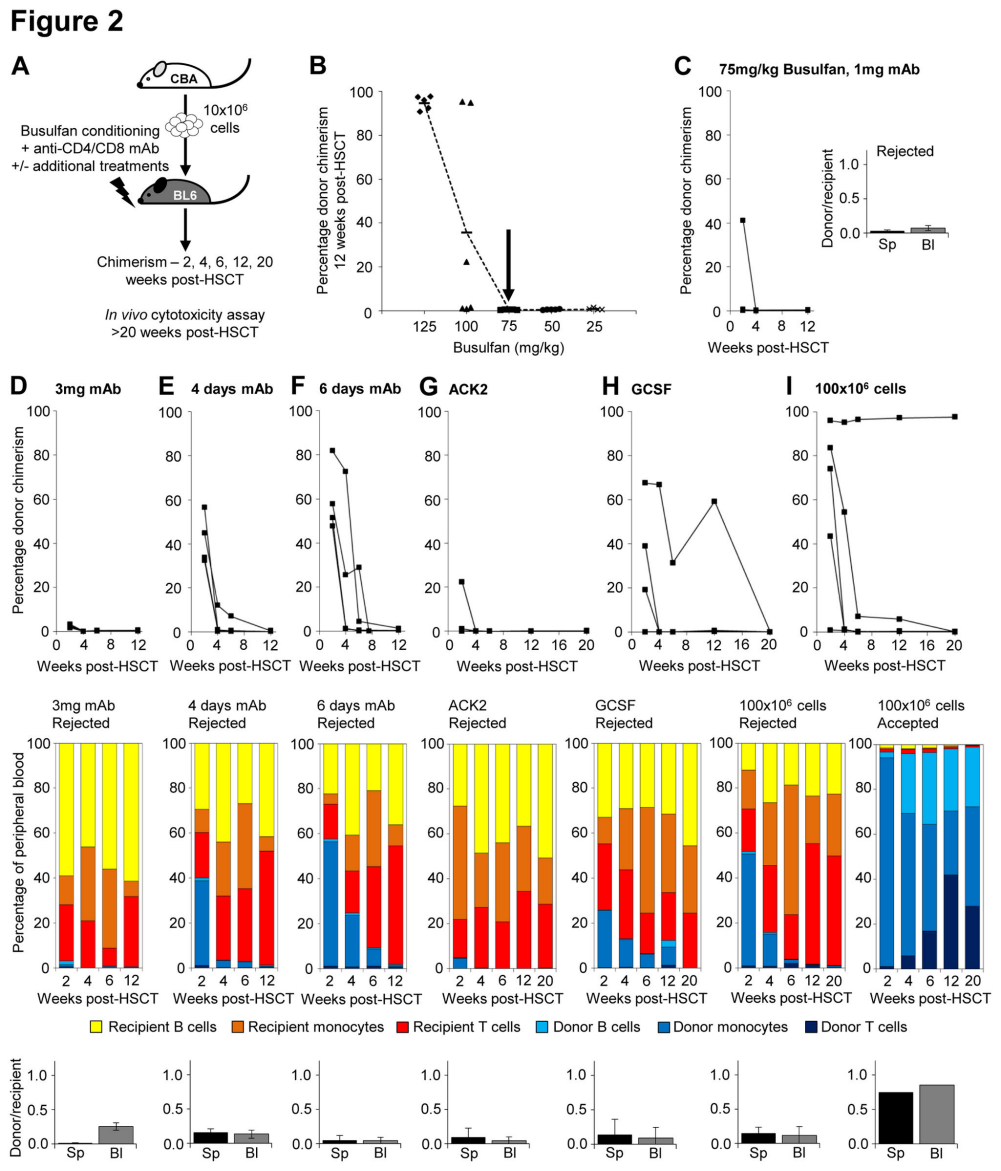
Increasing initial allogeneic chimerism is not sufficient for long-term engraftment with reduced intensity conditioning. (**A**) HSCT recipients (C57BL/6) were treated with busulfan conditioning before transplant with 10x10^6^ allogeneic (CBA) cells, and received 1mg anti-CD4 and anti-CD8 mAb on days 0, 2 and 4 relative to transplant with or without additional treatments as specified (n=4-6). (**B**) Donor chimerism at 12 weeks post-transplant was measured using flow cytometry in mice that had received 25-125mg/kg busulfan plus 1mg anti-CD4 and anti-CD8 mAb on days 0, 2 and 4. (**C**) Donor chimerism over time is shown for recipients of 75mg/kg busulfan and 1mg mAb. Tolerance to donor cells at >20 weeks post-transplant, measured using the *in* *vivo* cytotoxicity assay, is presented as ratio of donor: recipient splenocytes remaining in spleen (Sp) and blood (Bl) 20 hours after injection. Tolerance is indicated by values >0.6. Additional treatments tested in combination with this regimen included (**D**) an increased dose of 3mg anti-CD4 and anti-CD8 mAb (n=5), (**E**) 1mg of each mAb on days -1, 0, 2 and 4 (n=4), (**F**) 1mg on days -1 to 4 (n=4), (**G**) 500ug ACK2 treatment on day -7 (n=5), (**H**) 8ug G-CSF for 4 days before HSCT (n=5), or (**I**) a cell dose of 100x10^6^ cells (n=5). Donor chimerism in peripheral blood at 2, 4, 6, 12 and 20 weeks post-transplant, the mean percentage contribution of donor and recipient T cells, monocytes and B cells in peripheral blood at these time points, and *in* *vivo* cytotoxicity assay results from >20 weeks post-transplant are displayed. Where some mice accepted transplant and others rejected, the contribution of different lineages and the *in* *vivo* cytotoxicity results are separated for the mice with and without donor chimerism.

To compare RIC regimens in a clinically relevant setting, we first characterised a mouse model of allo-transplant across a full MHC barrier using clinically relevant cell doses that require both myeloablation and immunosuppression to achieve engraftment. We surmised that transplant from fully MHC mismatched CBA (H2D^k^) or Balb/c (H2D^d^) donors to C57BL/6 recipients (H2D^b^) would be appropriate as C57BL/6 recipients have increased stem cell numbers [32], and are reportedly resistant to transplant tolerance induction protocols [5,33]. MAC with 125mg/kg busulfan and transplant with 10x10^6^ cells from either CBA or Balb/c donors led to rejection in all C57BL/6 recipients, and at 2 weeks post-transplant 70-80% of the peripheral blood was recipient T cells, demonstrating a large T cell response to the allogeneic cells (Figures S2A,B). We therefore included immune suppression, adding non-depleting anti-CD4 and anti-CD8 mAbs at days 0, 2 and 4 (Figure 2A). Anti-CD4 and anti-CD8 mAbs are used together because CD4 and CD8 T cells can mediate HSCT rejection independently [34]. We chose non-depleting mAbs because they allow generation of T regulatory cells (Tregs) [11,34,35], which are important in generating transplant tolerance. Combining non-depleting anti-CD4 and anti-CD8 mAbs with 125mg/kg busulfan generates full engraftment in C57BL/6 recipients with 10x10^6^ CBA cells, but does not allow significant reduction of the busulfan dose (Figure 2B). A mixed response is observed with 100mg/kg busulfan, with 3/7 mice developing long-term engraftment, whilst rejection is observed in all recipients when busulfan is reduced to 75, 50 or 25mg/kg (Figure 2B). 

We therefore chose 75mg/kg busulfan for use in further RIC regimens as a dose that was just insufficient to attain engraftment in combination with 1mg anti-CD4 and anti-CD8 antibodies on days 0, 2 and 4 (Figure 2C). Increased or extended mAb treatments were tested, to ascertain if the immunosuppressive effect of anti-CD4/8 could be improved. Treatment with 3mg each mAb on days 0, 2 and 4 did not improve engraftment or tolerance (Figure 2D). Extension of treatment to 1mg each mAb on days -1, 0, 2 and 4, or every day from -1 to 4, improved initial engraftment to 30-80% at 2 weeks post-transplant (Figures 2E,F), but by 12 weeks post-transplant all mice had no donor chimerism. Improved initial engraftment did not lead to tolerance once chimerism was lost (Figures 2E,F). 

Given our findings in syngeneic transplant, that donor: recipient cell ratio in the BM niche plays a key role in attaining high donor engraftment, we compared regimens designed to improve donor: recipient cell number. Anti-ckit mAb, ACK2, was used to disrupt recipient stem cell niche interactions prior to HSCT, but had no effect on engraftment (Figure 2G). The use of GSCF to stimulate stem cell proliferation and egress from the BM niche prior to HSCT improved initial engraftment in some recipients, but lasting engraftment and tolerance were not observed (Figure 2H). A high cell dose (100x10^6^ cells), increasing the potential number of donor cells reaching the niche, allowed early engraftment in 4/5 recipients, but only 1 retained long-term donor chimerism and gained tolerance to donor cells (Figure 2I). This suggests that increasing the proportion of donor: recipient cells in the niche has some benefit, but is not sufficient to reliably overcome the immune response in MHC mismatched HSCT. 

In all these unsuccessful RIC regimens, any initial donor chimerism present at 2 weeks post-transplant consisted mostly of CD11b+ monocytes, whilst the contribution of CD3+ T cells and CD19+ B cells to the donor population increased gradually over time only in the single recipient of 100x10^6^ cells that retained donor chimerism (Figure 2D-I). In all regimens the percentage of recipient T cells is reduced compared to that observed in mice that received allotransplant following busulfan conditioning without mAb treatment (Figure 2D-I, Figure S2B), though not sufficiently to prevent graft rejection, except in a single mouse with very high initial donor chimerism (Figure 2I).

### Combined signal 1 and 2 T cell blockade is required for long-term allogeneic BM engraftment with NMC

**Figure 3:**
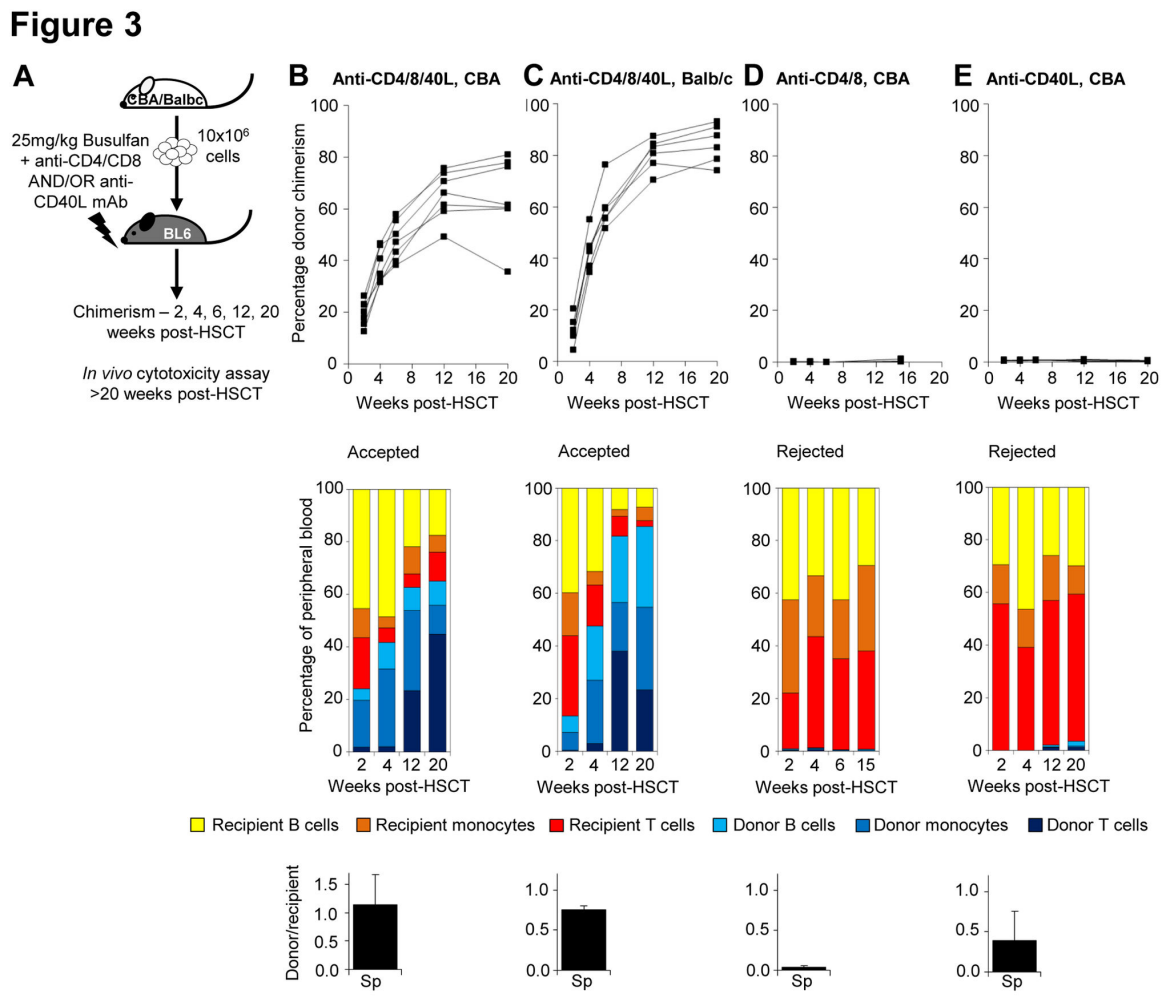
Combined signal 1 and 2 T cell blockade is required for long-term allogeneic engraftment with non-myeloablative conditioning. (**A**) HSCT recipients (C57BL/6) were treated with 25mg/kg busulfan and given allogeneic HSCT with anti-CD4/CD8, anti-CD40L, or combined mAb treatment. Donor chimerism and the mean percentage contribution of donor and recipient T cells, monocytes and B cells in peripheral blood at 2-20 weeks post-transplant, and the *in* *vivo* cytotoxicity assay results from >20 weeks post-transplant are displayed. Transplant acceptance was observed in recipients of 10x10^6^ (**B**) CBA donor cells (n=5), or (**C**) Balb/c donor cells (n=6), along with 1mg anti-CD4, anti-CD8 and anti-CD40L mAb on days 0, 2 and 4. Transplant rejection was observed in recipients of 10x10^6^ CBA donor cells with either (**D**) 1mg anti-CD4 and anti-CD8 mAb (n=5) or (**E**) 1mg anti-CD40L (n=6) on days 0, 2 and 4.

Following the failure of RIC regimens based on increasing the ratio of donor: recipient cells in the BM, we attempted to determine a successful RIC regimen that included additional immune suppression via costimulatory blockade. In mice that received 75mg/kg busulfan, anti-CD4/CD8 mAb treatment, plus 1mg of anti-CD40L to block costimulation, transplant with 10x10^6^ CBA cells generated long-term engraftment and tolerance in 4/5 recipients (Figures S3A,B). We then examined whether engraftment of 10x10^6^ CBA or Balb/c cells could also be achieved in C57BL/6 recipients using NMC of 25mg/kg of busulfan combined with anti-CD4/CD8/CD40L mAb (Figure 3A). We generated long-term donor engraftment and tolerance to donor splenocytes in recipients of CBA and Balb/c cells, achieving an average of 65% and 85% donor chimerism respectively by 20 weeks post-transplant (Figures 3B,C). As observed in syngeneic transplant, the donor population at 2 weeks post-transplant consists mainly of monocytes, but the contribution of donor T cells and B cells increases over time (Figures 3B,C). The recipient T cell population in successful engraftment does not appear to be significantly smaller than in the unsuccessful regimens at 2 weeks post-transplant, but decreases over time as donor chimerism is established (Figures 3B,C).

To determine whether combination of signal 1 and signal 2 T cell blockade was necessary for the success of this NMC regimen, C57BL/6 recipients were treated with 25mg/kg busulfan and either anti-CD4/CD8 mAb or anti-CD40L mAb (Figure 3A). A transplant of 10x10^6^ CBA cells was fully rejected by 2 weeks post-transplant in recipients of either signal 1 (Figure 3D) or signal 2 blockade alone (Figure 3E). The percentage of recipient T cells is reduced with either treatment, compared to that in mice receiving allotransplant following busulfan conditioning alone, but not sufficiently to allow graft acceptance (Figures 3D,E). Therefore the combination of these two treatments is essential for non-myeloablative transplant success.

**Figure 4:**
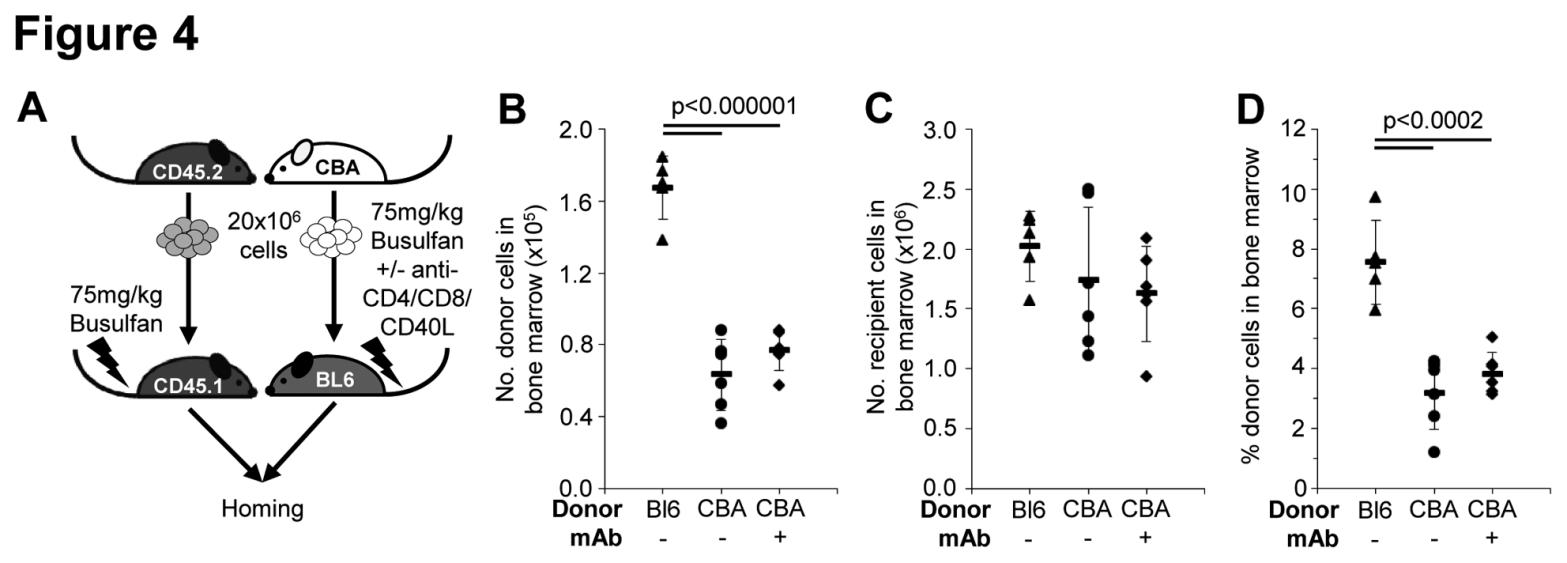
The success of allogeneic HSCT with combined signal 1 and 2 blockade is not due to increased homing of donor cells to the bone marrow. (**A**) HSCT recipients (C57BL/6) were treated with 75mg/kg busulfan and then received syngeneic transplant (n=6), allogeneic transplant (n=6), or allogeneic transplant plus 1mg anti-CD4, anti-CD8 and anti-CD40L mAb on the day of transplant (n=6). (**B**) The number of donor cells, (**C**) number of recipient cells, and (**D**) percentage of donor cells in bone marrow were determined 36 hours after transplant. Each data point represents one recipient, horizontal bars are the mean and error bars represent the standard deviation.

To determine whether combined signal 1 and 2 T cell blockade improved transplant success by having an effect on the donor: recipient cell ratio in BM, we compared homing in mice that all received 75mg/kg busulfan with either syngeneic HSCT (successful engraftment), allogeneic HSCT (rejection), or allogeneic HSCT with anti-CD4/CD8/CD40L mAb treatment (successful engraftment) (Figure 4A). The number of donor cells in BM is significantly reduced after allogeneic HSCT compared to syngeneic HSCT, but addition of mAb treatment, which ultimately results in transplant acceptance, does not increase homing to BM after allo-transplant (Figure 4B). There is no significant difference in the number of recipient cells in the BM after syngeneic or allogeneic transplant (Figure 4C), therefore the percentage of donor cells in BM after allo-transplant with or without mAb treatment is also significantly reduced compared to syngeneic transplant (Figure 4D). This confirms that the number of donor cells migrating to BM is less critical than blocking the immune response in allogeneic transplant.

## Discussion

Many murine RIC HSCT regimens use high cell doses, but in the clinic patients often receive a dose only just sufficient to reconstitute the haematopoietic system [25]. Using cell doses that better reflect the equivalent cell dose a patient may receive, we found that syngeneic engraftment could be achieved using a non-myeloablative dose of 25mg/kg busulfan, increasing over time to up to 80% in recipients of 10x10^6^ cells. This gradual increase has been observed by others; in syngeneic HSCT with 20mg/kg busulfan and 20-28x10^6^ cells, low initial engraftment increased to 70-90% by 12-17 weeks post-transplant [13,36]. This may be because low dose busulfan leads to delayed suppression of peripheral blood counts, reaching a maximum 2-3 weeks after treatment [13,36]. Unlike irradiation, busulfan suppresses HSCs and progenitors by an apoptosis-independent mechanism, leading to a decreased frequency of colony forming cells and reduced colony size from BM taken 7 days after busulfan treatment [37,38]. This probably means that busulfan treated cells are not removed immediately but are disadvantaged in comparison to transplanted cells, allowing gradual engraftment as donor cells out-compete damaged recipient cells. However, in allogeneic transplant the immune response against donor cells means they are unable to out-compete recipient cells without full myeloablation and immune suppression. 

Studies of RIC HSCT have shown that Tregs are important in generation, but not maintenance, of tolerance [17,39]. Therefore in our allogeneic HSCT model we used non-depleting T cell blocking mAbs against CD4 and CD8, which allow generation of Tregs [11,34,35], and are used together because CD4 and CD8 T cells can independently mediate HSCT rejection [34]. Non-depleting anti-CD4/CD8 mAbs overcome allogeneic HSCT rejection in combination with 125mg/kg busulfan, but myeloablation cannot be reduced. Others have reported that agents inhibiting alloreactive CD4 and CD8 T cell proliferation do not prevent HSCT rejection, and are not as potent as anti-CD40L costimulation blockade in allowing HSCT acceptance [26]. Using 200x10^6^ cells, costimulation blockade generates mixed chimerism [10], but T cell depleting mAbs against CD4 and CD8 do not [7]. It has been suggested that costimulation blockade is better than T cell depletion at overcoming intrathymic alloresistance [7], and this may extend to non-depleting T cell blockade as we found it necessary to add anti-CD40L mAb when busulfan dose was reduced. However anti-CD40L only generates CD4 T cell tolerance and cannot overcome CD8 T cell mediated graft rejection [39-41], which is important in human transplant acceptance. We determined that costimulation blockade alone was not sufficient for allo-transplant success with NMC, and the importance of including CD4/CD8 blockade is also demonstrated by the higher levels of engraftment we achieve compared to regimens using low dose myeloablation and anti-CD40L mAb alone, despite their using 2-4 fold higher cell doses [15,17,18,21]. We have also demonstrated that whilst a variety of RIC regimens were able to reduce the post-transplant expansion of recipient T cells, this was not sufficient to prevent graft rejection, except in the regimen that combined anti-CD40L with anti-CD4/CD8 mAbs. This combination of CD4/8 blockade with costimulation blockade allowed a gradual increase in donor cells of multiple lineages over time, in a similar way to that observed in syngeneic transplant.

Myeloablation with irradiation has been reported to increase SDF-1 production in BM [42]; however in contrast, we observed a reduction of SDF-1 in BM of recipients treated with increasing doses of busulfan. Busulfan is delivered over a number of days, therefore mice receiving higher doses will have significant cell death and fewer cells remaining in their BM before transplant. High dose busulfan may induce individual cells to produce more SDF-1, but reduced cell number means that overall SDF-1 levels are lower. With high dose busulfan, the number of syngeneic donor cells that home to BM is also reduced, correlating with the SDF-1 gradient, and reduced homing has also been observed in irradiated compared to non-irradiated mice [43]. However, long-term donor chimerism is lower after syngeneic HSCT with 25mg/kg compared to 125mg/kg Busulfan, therefore the number of cells homing to BM is clearly not as important as achieving a high percentage of donor cells compared to recipient cells. We therefore attempted to increase the ratio of donor: recipient cells in the BM of allogeneic HSCT recipients treated with 75mg/kg busulfan and non-depleting anti-CD4/CD8 mAb. However, extended mAb treatment, high cell dose, or treatment with G-CSF did not lead to long-term engraftment, despite increasing initial engraftment. G-CSF has been used to improve engraftment via transient mobilisation of stem cells out of the niche, but in syngeneic rather than allogeneic transplant, using 160cGy irradiation [28]. ACK2 has also been used to deplete HSCs from the BM niche, allowing 16-90% donor chimerism in immune deficient recipients [27]. It is likely that these approaches were unsuccessful in our model as the immune response could not be overcome by increasing the ratio of donor: recipient HSCs in the BM niche. This is supported by our observations that homing is significantly reduced in allogeneic compared to syngeneic HSCT, but that treatment with anti-CD4/CD8/CD40L mAb, which allows allo-engraftment, does not increase homing in allogeneic recipients. Thus improved homing is not essential, and improving early engraftment in the BM niche is unlikely to be sufficient for non-myeloablative HSCT without further immune suppression.

Addition of anti-CD40L costimulatory blockade to anti-CD4/CD8 mAb treatment allows reduction of busulfan to 25mg/kg. Anti-CD40L mAb is widely used in mouse models of RIC allogeneic HSCT, but it causes perturbation of blood coagulation and thromboembolic effects in non-human primates and humans [44]. However, its inclusion is acceptable as alternative forms of anti-CD40L mAb lacking the problematic epitope are as effective [6], whilst anti-CD40 mAbs are also in development [45]. The combination of anti-CD4, anti-CD8 and anti-CD40L mAb has also been successful in murine allo-transplant without myeloablation; however the protocol of Graca et al only works with certain strain combinations and was less effective in C57BL/6 syngeneic transplant than in CBA allogeneic transplant [5]. C57BL/6 mice are reportedly resistant to CD4/CD8 mAb tolerance induction protocols [33], possibly because C57BL/6 stem cells are more abundant than other mouse strains [32], but we overcome this resistance by adding non-myeloablative busulfan. Anti-CD4/CD8, or anti-CD40L mAb do not generate allogeneic HSCT acceptance between strains with major and minor MHC mismatches without some myeloablation [4], but we agree that in combination these mAbs work synergistically, as reported by Graca et al [5]. In their model, low donor chimerism develops in permissive strains treated with mAb, whilst we achieve higher donor chimerism in non-permissive strains treated with mAb and non-myeloablative busulfan. 

The RIC regimen developed by Graca et al also includes pre-treatment of recipients with anti-CD4/CD8 mAb 4 weeks before transplant, which increases memory and regulatory cells [5]. Recipients that received no pre-treatment underwent a protocol that closely resembles our own but generated no donor chimerism [5]; therefore addition of 25mg/kg busulfan appears essential. Addition of non-myeloablative busulfan can greatly reduce the cell dose required for allogeneic engraftment. With T cell depleting mAbs and sirolimus, addition of 5-40mg/kg busulfan allowed a 6-fold reduction in the required cell dose [16], and with costimulation blockade addition of 20mg/kg busulfan allowed a 100-fold reduction in cell dose [39]. Our use of 25mg/kg busulfan with signal 1 and 2 T cell blockade allows a clinically relevant cell dose to generate higher donor chimerism than many other protocols. When the same antibodies are used with a mAb pre-treatment stage rather than myeloablation, just 25-40% donor chimerism is achieved using 2-4 fold higher cell doses [5,6,11]. Compared to protocols with 3Gy irradiation (which has a similar effect on white blood cells to 20mg/kg busulfan [39]) and anti-CD40L mAb we also achieve slightly higher chimerism with fewer cells [15,17,22,46]. The only protocol that achieves similar levels of chimerism involves depleting mAbs, 20-40mg/kg busulfan, sirolimus and 25-40x10^6^ cells [15,16]. However, the use of non-depleting rather than depleting mAb is preferable for Treg generation, and there is evidence that sirolimus can boost T cell responses in some circumstances [47]. 

In conclusion we have used a stringent mouse model of allogeneic HSCT that requires full myeloablation and immune suppression for engraftment, to compare RIC regimens. We have determined that although in syngeneic transplant increasing the ratio of donor: recipient cells in the BM niche positively influences engraftment, improvements in initial engraftment, either by creating more space in the BM niche, or by delivering more donor cells, are ineffective in RIC for allo-transplant. To achieve long-term high donor chimerism in allogeneic transplant using clinically relevant cell doses and a non-myeloablative busulfan dose requires immune blockade, of T cell activation via both signal 1 and 2. Neither signal 1 or 2 blockade alone was sufficient to achieve allotransplant acceptance thus highlighting the importance of T cell signalling in the transplant setting. Addition of costimulatory blockade was critical for reduction of busulfan doses, which is informative for the clinic, where CTLA4Ig (Abatacept/Belatacept) is already approved for costimulatory blockade [48] but is not currently used in HSCT regimens. We would predict that once T cell responses are effectively blocked during allo-transplant, the factors used to achieve improved donor: recipient numbers in the BM niche could then be used to achieve maximal donor chimerism.

## Materials and Methods

### Mice

CBA (H2D^k^) and Balb/c (H2D^d^) (Harlan, UK), C57BL/6 (CD45.2, H2D^b^) and B6.SJL-*Ptprc* ^*a*^ *Pepcb*/BoyJ (CD45.1, H2D^b^) mice were maintained at the University of Manchester Biological Services Unit, in a 12/12h light/dark cycle and constant temperature with access to food and water *ad libitum*. All animal procedures were approved by the University of Manchester Ethical Review Process Committee, and carried out in accordance with the Animals (Scientific Procedures) Act, 1986 (UK) and Home Office licence PPL40/3117.

### Haematopoietic stem cell transplant

#### Myeloablation

C57BL/6 recipients (6-12 weeks old) (n=3-7) received conditioning regimens. MAC used 10Gy TBI (2 x 5Gy separated by 3-4 hours) on the day of transplant, or 125mg/kg busulfan (Busilvex 6mg/ml, Pierre Fabre), (5 x 25mg/kg/day over 5 days) via intraperitoneal (IP) injection finishing on day -1 to transplant [49-51]. RIC was achieved by reducing busulfan to 50, 75 or 100mg/kg (25mg/kg/day). NMC used one dose of 25mg/kg busulfan. Recipients received acidified water (pH 2.8) and irradiated food during the period of immune compromise following conditioning, and dietary supplementation with mash and jelly to improve post-transplant weight outcomes. 

#### Immune blockade

Some groups received non-depleting mAbs that block CD4 (YTS177), CD8 (YTS105) and CD40L (MR1), produced from hybridoma cell lines kindly provided by Prof. H. Waldmann [34,40]. The protocol for mAb production was modified from Honey et al [40]. Cells were cultured in CELLine CL 1000 flasks (Integra), in Iscove’s Modified Dulbecco’s Medium (IMDM) with 5% foetal calf serum (FCS). Fractionated ammonium sulphate precipitation was used to purify mAb from the cell culture supernatant. The mAb solutions were dialysed into phosphate buffered saline (PBS), concentrated using VivaSpin centrifugal concentration columns (Sartorius Stedim Biotech), and concentration estimated using absorbance at 280nm. SDS and native polyacrylamide gel electrophoresis were used to test for purity and denaturation respectively.

Standard mAb treatment was 1mg each of anti-CD4 and anti-CD8 via IP injection on days 0, 2 and 4 relative to transplant, with additional use as stated. Baytril oral solution, (0.25mg Enrofloxacin/ml) was given for the duration of mAb treatment and 1 week afterwards to avoid infections.

#### Additional factors

Additional factors were tested in combination with 25 or 75mg/kg busulfan and anti-CD4/CD8 mAb treatment; 500μg ACK2 (eBioscience) on day -7 via intravenous (IV) injection [27], 4μg G-CSF (Neupogen, Amgen) twice daily for 4 days before and once on the day of transplant via subcutaneous (SC) injection [28], or 1mg anti-CD40L on days 0, 2 and 4.

#### Transplant

Donor BM was isolated as previously described [52]. Donor cells were delivered via tail vein injection.

### Donor chimerism

Haematopoietic chimerism was quantified by measuring the percentage of donor: recipient cells in peripheral blood (PB), BM or spleen using flow cytometry (FACS Canto II, FACS Diva). Donor and recipient cells were distinguished using antibodies (BD Biosciences) against CD45.1, CD45.2, H2D^k^ or H2D^b^ as relevant, and lineage staining against CD19 (B cells), CD11b (monocytes) and CD3 (T cells) in 5% solutions of ToPro3 iodide (Invitrogen). 

### In vivo cytotoxicity assay

The *in vivo* cytotoxicity assay was modified from Yamazaki et al [24]. Splenocytes from donor and recipient (1x10^7^) were stained with 10µM Celltracker Orange CMTMR or 5µM Celltrace CFSE (Invitrogen), according to the manufacturer’s instructions, washed, mixed in a 1:1 ratio and delivered via IV injection to transplant recipients at >20 weeks post-transplant. After 20 hours, PB and splenocytes were collected and the donor (CFSE) and recipient (Celltracker Orange) ratio determined using flow cytometry. Tolerised recipients retain close to a 1:1 mix of donor and recipient stained splenocytes, whilst donor rejection is indicated by the reduction or absence of stained donor splenocytes. Results are presented as percentage of donor cells/percentage of recipient cells; >0.6 indicates tolerance, <0.6 indicates non-tolerance.

### SDF-1α ELISA

PB collected with sodium citrate anti-coagulant was centrifuged at 1000g to isolate plasma. BM was prepared [52], then centrifuged at 300g and the supernatant retained for analysis. SDF-1α levels were tested using the Quantikine mouse CXCL12/SDF-1α immunoassay (R&D Systems) following manufacturer’s instructions. 

### Statistical analysis

Statistical significance was determined using one-way ANOVA, applying Tukey’s multiple comparisons test and assuming significance where p<0.05 of a studentised range of Q. 

## Supporting Information

Figure S1 **In syngeneic transplant, conditioning with 125mg/kg busulfan or 10Gy irradiation leads to equivalent long-term chimerism.** (**A**) HSCT recipients (CD45.1) were conditioned with 125mg/kg busulfan (BU) or 10Gy total body irradiation (TBI) before receiving 2x10^6^ syngeneic (CD45.2) donor bone marrow cells (n=5). (**B**) Mean percentage donor chimerism in peripheral blood over time is shown; error bars represent standard deviation.
(TIF)

Figure S2 **In allogeneic transplant, 125mg/kg busulfan is insufficient for engraftment.** (**A**) HSCT recipients (C57BL/6) were treated with 125mg/kg busulfan before allogeneic HSCT with 10x10^6^ CBA (n=3) or Balb/c (n=6) donor bone marrow cells. (**B**) The mean percentage contribution of donor and recipient T cells, monocytes and B cells to peripheral blood at 2 weeks post-transplant is displayed.
(TIF)

Figure S3 **Combined signal 1 and 2 T cell blockade allows long-term allogeneic engraftment with reduced intensity conditioning.** (**A**) HSCT recipients (C57BL/6) were treated with 75mg/kg busulfan before transplant with 10x10^6^ CBA donor cells, along with along with 1mg anti-CD4, anti-CD8 and anti-CD40L mAb on days 0, 2 and 4 (n=5). (**B**) Donor chimerism in peripheral blood and the mean percentage contribution of donor and recipient T cells, monocytes and B cells to peripheral blood at 2-20 weeks post-transplant, and the *in* *vivo* cytotoxicity assay results from >20 weeks post-transplant are displayed. The contribution of different lineages to peripheral blood and the *in* *vivo* cytotoxicity results are separated for the mice with and without donor chimerism.
(TIF)
