# Supplementary figures and images for: Signal One and Two Blockade Are Both Critical for Non-Myeloablative Murine HSCT across a Major Histocompatibility Complex Barrier
Source: PLoS One. 2013 Oct 17;8(10):e77632. doi: 10.1371/journal.pone.0077632 (PMC3798400; doi:10.1371/journal.pone.0077632)

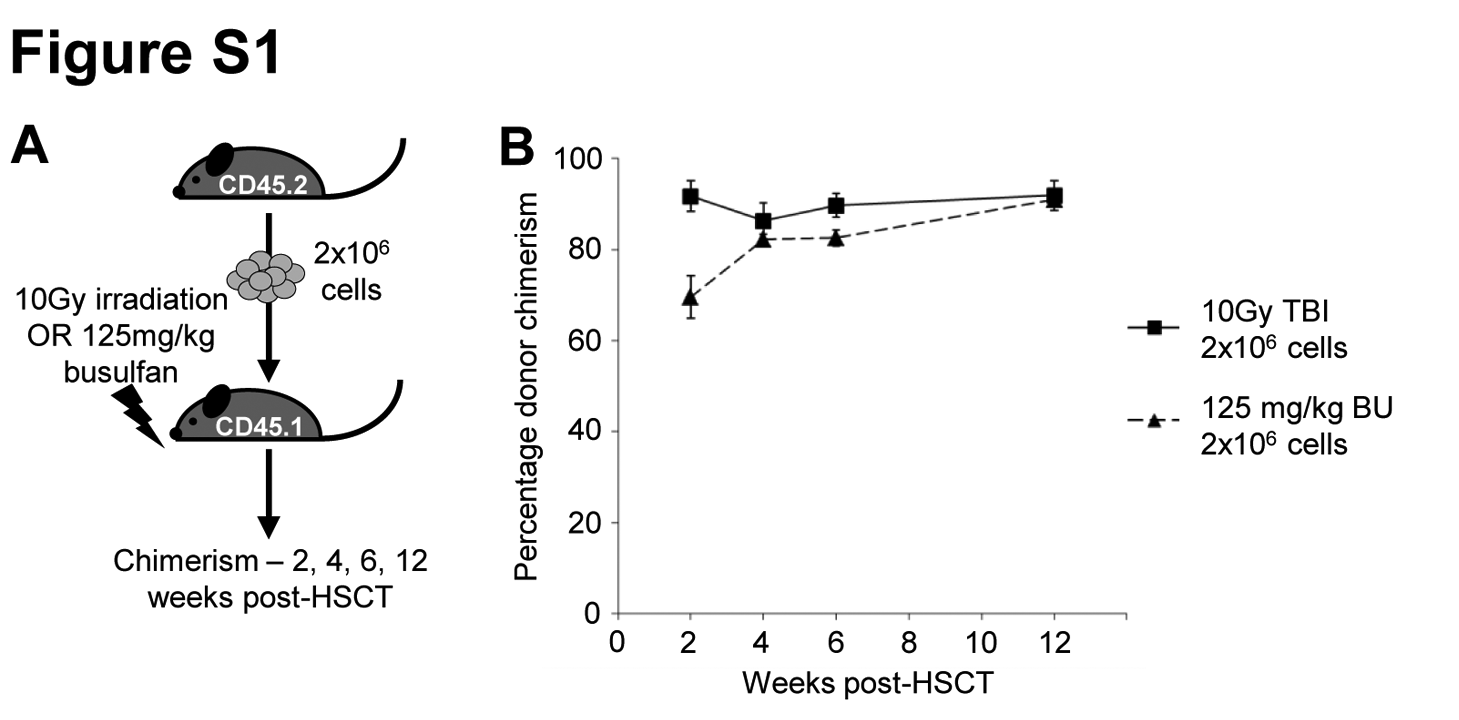

Supplement: Figure S1 — In syngeneic transplant, conditioning with 125mg/kg busulfan or 10Gy irradiation leads to equivalent long-term chimerism. (A) HSCT recipients (CD45.1) were conditioned with 125mg/kg busulfan (BU) or 10Gy total body irradiation (TBI) before receiving 2x10^6^ syngeneic (CD45.2) donor bone marrow cells (n=5). (B) Mean percentage donor chimerism in peripheral blood over time is shown; error bars represent standard deviation. (TIF) [file pone.0077632.s001.tif]

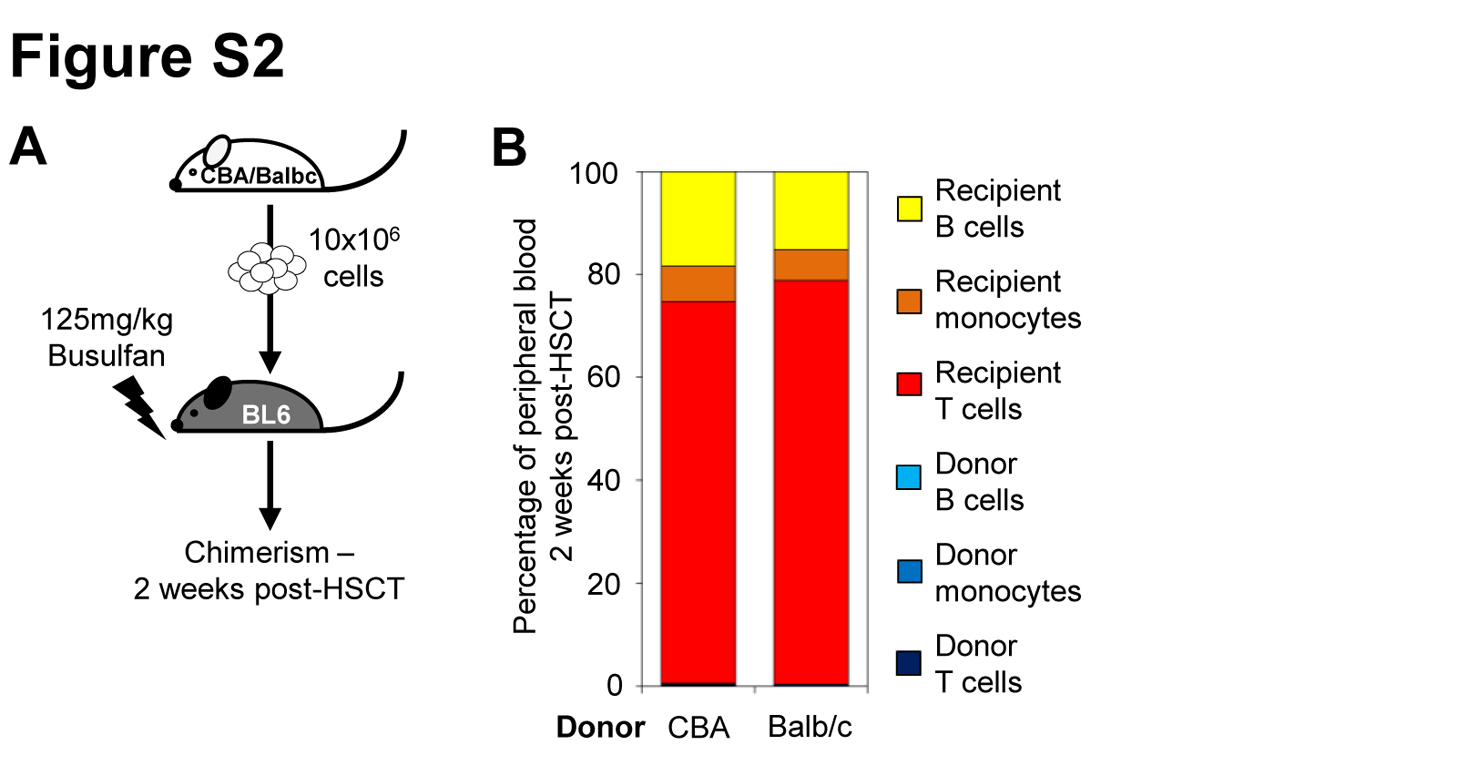

Supplement: Figure S2 — In allogeneic transplant, 125mg/kg busulfan is insufficient for engraftment. (A) HSCT recipients (C57BL/6) were treated with 125mg/kg busulfan before allogeneic HSCT with 10x106 CBA (n=3) or Balb/c (n=6) donor bone marrow cells. (B) The mean percentage contribution of donor and recipient T cells, monocytes and B cells to peripheral blood at 2 weeks post-transplant is displayed. (TIF) [file pone.0077632.s002.tif]

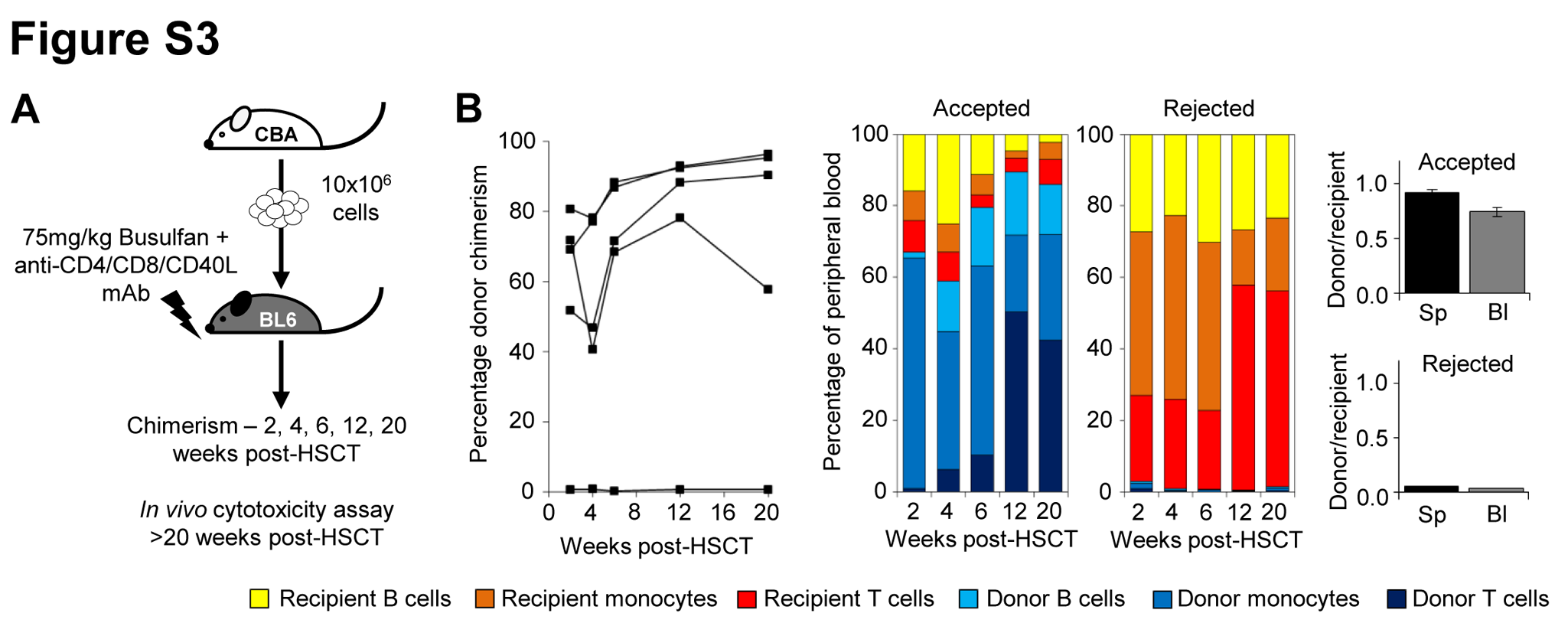

Supplement: Figure S3 — Combined signal 1 and 2 T cell blockade allows long-term allogeneic engraftment with reduced intensity conditioning. (A) HSCT recipients (C57BL/6) were treated with 75mg/kg busulfan before transplant with 10x106 CBA donor cells, along with along with 1mg anti-CD4, anti-CD8 and anti-CD40L mAb on days 0, 2 and 4 (n=5). (B) Donor chimerism in peripheral blood and the mean percentage contribution of donor and recipient T cells, monocytes and B cells to peripheral blood at 2-20 weeks post-transplant, and the in vivo cytotoxicity assay results from >20 weeks post-transplant are displayed. The contribution of different lineages to peripheral blood and the in vivo cytotoxicity results are separated for the mice with and without donor chimerism. (TIF) [file pone.0077632.s003.tif]
